# Arabidopsis PCaP2 Functions as a Linker Between ABA and SA Signals in Plant Water Deficit Tolerance

**DOI:** 10.3389/fpls.2018.00578

**Published:** 2018-05-08

**Authors:** Xianling Wang, Yu Wang, Lu Wang, Huan Liu, Bing Zhang, Qijiang Cao, Xinyu Liu, Shuangtian Bi, Yanling Lv, Qiuyang Wang, Shaobin Zhang, Ming He, Shuang Tang, Shuo Yao, Che Wang

**Affiliations:** ^1^College of Biological Science and Biotechnology, Shenyang Agricultural University, Shenyang, China; ^2^Department of Medicine, HE University School of Clinical Medicine, Shenyang, China; ^3^Vegetable Research Institute of Liaoning Academy of Agricultural Sciences, Shenyang, China

**Keywords:** PCaP2, water deficit, ABA, SA, SnRK2, PR, Arabidopsis

## Abstract

Water stress has a major influence on plant growth, development, and productivity. However, the cross-talk networks involved in drought tolerance are not well understood. Arabidopsis PCaP2 is a plasma membrane-associated Ca^2+^-binding protein. In this study, we employ qRT-PCR and β-glucuronidase (GUS) histochemical staining to demonstrate that *PCaP2* expression was strongly induced in roots, cotyledons, true leaves, lateral roots, and whole plants under water deficit conditions. Compared with the wild type (WT) plants, *PCaP2*-overexpressing (*PCaP2*-OE) plants displayed enhanced water deficit tolerance in terms of seed germination, seedling growth, and plant survival status. On the contrary, *PCaP2* mutation and reduction via *PCaP2*-RNAi rendered plants more sensitive to water deficit. Furthermore, *PCaP2*-RNAi and *pcap2* seedlings showed shorter root hairs and lower relative water content compared to WT under normal conditions and these phenotypes were exacerbated under water deficit. Additionally, the expression of *PCaP2* was strongly induced by exogenous abscisic acid (ABA) and salicylic acid (SA) treatments. *PCaP2*-OE plants showed insensitive to exogenous ABA and SA treatments, in contrast to the susceptible phenotypes of *pcap2* and *PCaP2*-RNAi. It is well-known that SNF1-related kinase 2s (SnRK2s) and pathogenesis-related (PRs) are major factors that influence plant drought tolerance by ABA- and SA-mediated pathways, respectively. Interestingly, PCaP2 positively regulated the expression of drought-inducible genes (*RD29A*, *KIN1*, and *KIN2*), ABA-mediated drought responsive genes (*SnRK2.2*, *-2.3*, *-2.6*, *ABF1*, *-2*, *-3*, *-4*), and SA-mediated drought responsive genes (*PR1*, *-2*, *-5*) under water deficit, ABA, or SA treatments. Taken together, our results showed that PCaP2 plays an important and positive role in Arabidopsis water deficit tolerance by involving in response to both ABA and SA signals and regulating root hair growth. This study provides novel insights into the underlying cross-talk mechanisms of plants in response to water deficit stress.

## Introduction

Water deficit is one of the most acute abiotic stresses affecting plant growth and the economic yield of crop plants. It leads to alterations in various cellular processes in plants, for example, gene expression, photosynthesis, protein synthesis, carbon partitioning, lipid metabolism, and osmotic homeostasis (Hua et al., 2012; Jarzyniak and Jasiński, 2014; Fleta-Soriano and Munné-Bosch, 2016). Phytohormones, such as abscisic acid (ABA), salicylic acid (SA), gibberellin (GA), indole-3-acetic acid (IAA), and jasmonic acid (JA) function as central factors that link and reprogram these complex cellular processes (Hua et al., 2012).

In response to water deficit, ABA, a well-known stress phytohormone, is rapidly induced, leading to the expression of stress-responsive genes and the activation of plants’ cellular physiological adaptation to water stress (Fujii and Zhu, 2009; Cutler et al., 2010; Weiner et al., 2010). In the ABA signaling pathway, SNF1-related kinase 2s (SnRK2s) are central regulators that mediate ABA-responsive transcription factors and genes to activate ABA-mediated physiological processes (Yoshida et al., 2002; Boudsocq et al., 2004; Fujita et al., 2009; Umezawa, 2009; Vlad et al., 2009; Raghavendra et al., 2010; Kulik et al., 2011; Ambrosone et al., 2015).

Among the 10 SnRK2s in Arabidopsis, SnRK2.2, SnRK2.3, and SnRK2.6 function as central regulators in response to ABA and drought. Genetic analysis has shown that the Arabidopsis triple mutant *snrk2.2*/*snrk2.3*/*snrk2.6* exhibits greatly reduced water deficit tolerance and is extremely insensitive to ABA (Fujii and Zhu, 2009; Fujita et al., 2009). The triple mutant is strongly impaired in ABA- and drought-responsive genes expression under water stress (Fujita et al., 2009). The phenotype of the triple mutant indicates that these three SnRK2s are partially redundant, although all of them are crucial for plants’ response to water stress and ABA, as well as ABA-mediated seed germination and dormancy. Seed dormancy, germination, and seedling growth of *snrk2.2*/*snrk2.3* mutants are greatly insensitive to exogenous ABA. In contrast, *snrk2.6* shows a significant increase in leaf water loss after ABA treatment (Yoshida et al., 2002; Fujii et al., 2007; Nakashima et al., 2009). This could be because SnRK2.6/ OST1 are important for stomatal movements, as they phosphorylate anion (SLAC1) and cation (KAT1) channels, which might be required for ABA-dependent stomatal closing in response to water deficit (Pilot et al., 2001; Geiger et al., 2009, 2010; Lee et al., 2009; Sato et al., 2009). In addition, guard cells (GCs) display transcriptional memory in a daily dehydration stress and watered recovery cycle. SnRK2.2, SnRK2.3, and SnRK2.6 have distinguishable roles in the process: SnRK2.2 and SnRK2.3 are more important for implementing guard cell stress memory, while SnRK2.6 is more important for overall stomatal control in the subsequent dehydration response (Virlouvet and Fromm, 2015). It has been found that SnRK2.2, -2.3, and 2.6 are regulated by nitric oxide (NO), phosphatidic acid (PA), and Ca^2+^ changes (Boudsocq et al., 2004; Fujii and Zhu, 2009; Kulik et al., 2011), suggesting SnRK2s may be regulated by complex pathways during water stress; however, such mechanisms remain largely unknown.

The phytohormone SA plays an important role in various plant developmental processes and responses to abiotic and biotic stress (Raskin, 1992; Bandurska and Stroiński, 2005; Khan et al., 2012a,b, 2013). Water deficit induces increased endogenous SA levels in various plants (Munne-Bosch and Penuelas, 2003; Miura and Tada, 2014). Exogenous treatment with SA modulates plant drought resistance through multiple pathways such as oxidative stress (Alam et al., 2013), stomatal conductance (Hao et al., 2010; Khokon et al., 2011; Habibi, 2012), antioxidant defense system (Hayat et al., 2008; Saruhan et al., 2012), and NO production (Hao et al., 2010; Khokon et al., 2011). Additionally, some SA-responsive genes are involved in plant response to water deficit, such as *GST1*, *GST2*, *GR*, and *MDHAR* in *Triticum aestivum* (Kang et al., 2013) and *MPK3*, *MPK4*, *MPK6*, *PR1*, *PR2*, and *PR5* in *Arabidopsis thaliana* (Ichimura et al., 2000; Ahlfors et al., 2004; Gudesblat et al., 2007; Liu P. et al., 2013; Liu W.X. et al., 2013). Some Arabidopsis mutants that accumulate endogenous SA (*adr1, acd6*, *cpr5 myb96-1d*, and *siz1*) show both SA-mediated disease resistance and water deficit tolerance (Miura et al., 2013). One genetic analysis reports that Arabidopsis seedlings overexpressing *PR1*, *PR2*, or *PR5* are drought tolerant (Liu W.X. et al., 2013). In addition, *PR1*, *-2*, and *-5* genes are widely used as marker genes for SA-mediated drought tolerance in plants. For example, both SA-accumulating mutants (*cpr5 and acd6*) and overexpression of transcription factor Di19 in Arabidopsis improve drought tolerance via SA-induced expression of *PR1*, *-2*, *-5* genes (Liu P. et al., 2013; Liu W.X. et al., 2013). Interestingly, SA treatments lead to an increase of ABA and proline in the barley leaves (Bandurska and Stroiński, 2005); however, the relationship between SA and ABA signals in water deficit remains unknown.

Arabidopsis microtubule-associated protein-18/plasma membrane-associated Ca^2+^-binding protein-2 (MAP18/PCaP2) is important for several physiological activities. For example, it is involved in Ca^2+^ binding and the organization of cortical microtubules (MTs) and F-actin. It also has a critical role in root hair, pollen tube growth, and directional cell growth (Wang et al., 2007; Kato et al., 2010, 2013; Zhu et al., 2013; Zhang et al., 2015; Kang et al., 2017). For example, the cell polarity and cortical microtubule array in line 2 of *MAP18*-overexpressing Arabidopsis (OE2) and line 18 of *MAP18* RNAi transgenic Arabidopsis (R18) are altered (Wang et al., 2007). The T-DNA insertion line *map18* (SALK_021652), which is confirmed as a knock-down mutant by qRT-PCR analysis, displays abnormal pollen tube growth and root hair growth (Kato et al., 2013; Zhu et al., 2013; Zhang et al., 2015; Kang et al., 2017).

Interestingly, the mRNA expression level of *PCaP2* is induced by heat, cold, drought, ABA, SA, osmotic stress, and GA3 (Kato et al., 2010, 2013). This implies that it may function in response to abiotic stress and phytohormone signals. Root hairs are the main sites of water absorption in plants, which is important for water deficit tolerance (Worrall and Roughley, 1976; Zahran and Sprent, 1986; Schnall and Quatrano, 1992). Thus, we hypothesized that PCaP2 might be an important regulator of plant water deficit tolerance in various pathways, such as phytohormone signals and root hair growth, suggesting that PCaP2 might be a cross-talker between complex mechanisms involved in plant water deficit tolerance. In this study, we found that PCaP2 is vital for plant water deficit tolerance by responding to ABA and SA signals, regulating the expression of the key ABA- and SA-mediated genes, and affecting root hair growth. Collectively, our data provide novel evidence of the underlying complex mechanisms, especially of crosstalk between ABA and SA signaling pathways in plant water deficit tolerance.

## Results

### The Expression of *PCaP2* Is Highly Induced in All Tissues in Water Deficit

**FIGURE 1:**
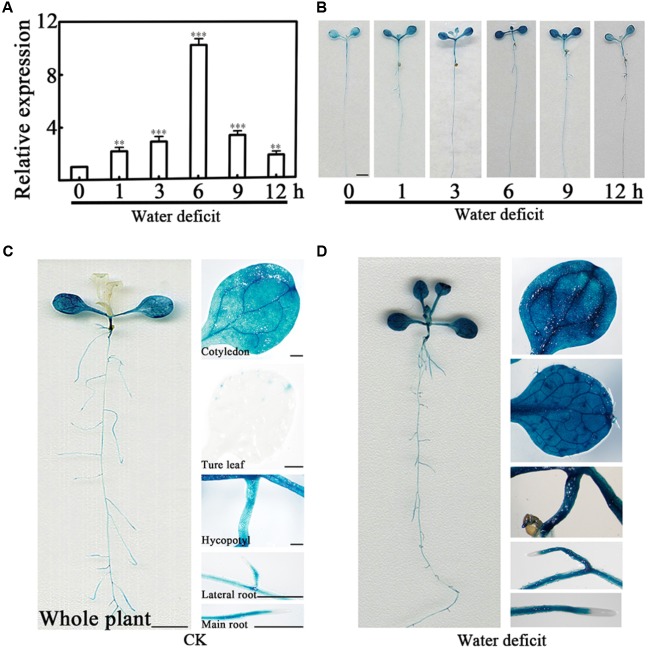
Expression pattern of *PCaP2* under water deficit stress. **(A)** Relative expression of *PCaP2* in response to water deficit. Fourteen-day-old WT seedlings were treated with water deficit for 0, 1, 3, 6, 9, and 12 h, and the expression of *PCaP2* was detected by qRT-PCR. Data represent mean values of three biological replicates ± SE. The significant difference was determined by ANOVA in comparison to 0 h: ^∗∗^*P* < 0.01, ^∗∗∗^*P* < 0.001. **(B)** Comparison of *PCaP2* expression levels treated with water deficit different times by GUS staining. Seven-day-old seedlings of p*PCaP2*::GUS transgenic plants were treated with water deficit for 0, 1, 3, 6, 9, and 12 h. Scale bar = 2.5 mm. **(C, D)** Analysis of the expression pattern of *PCaP2* by GUS staining of pPCaP2::GUS transgenic seedlings in normal (without water deficit treatment) **(C)** and water deficit treatments for 6 h **(D)**. At least 15 seedlings from each sample were used for every technical replicate and three biological replicates were conducted. The scale bar is 5 mm in the pictures of whole plant, and 0.25 mm in the pictures of cotyledons, true leaves, hypocotyls, lateral roots and main roots.

To fully understand the expression pattern of *PCaP2* under water deficit stress, we examined the expression of *PCaP2* in more details by quantitative real-time PCR (qRT-PCR) and β-glucuronidase (GUS) staining. Firstly, the wild type (WT) plants were exposed to dehydration conditions for 1, 3, 6, 9, and 12 h. The qRT-PCR results showed that *PCaP2* expression was highly induced by water deficit treatments from 1 to 12 h, with the peak level of 10-fold increased at 6 h treatment (**Figure 1A**). Furthermore, GUS staining showed that the promoter activity of *PCaP2* was significantly increased after water deficit treatments for 6 h which was consistent with the results of qRT-PCR (**Figure 1B**) and *PCaP2* expression was induced in primary roots and lateral roots, cotyledons, true leaves, and the whole seedlings after water deficit treatments (**Figures 1B–D**).

### *PCaP2*-OE Plants Display Increased Tolerance While *PCaP2* RNAi and Mutant Seedlings Are Hypersensitive in Response to Water Deficit

**FIGURE 2:**
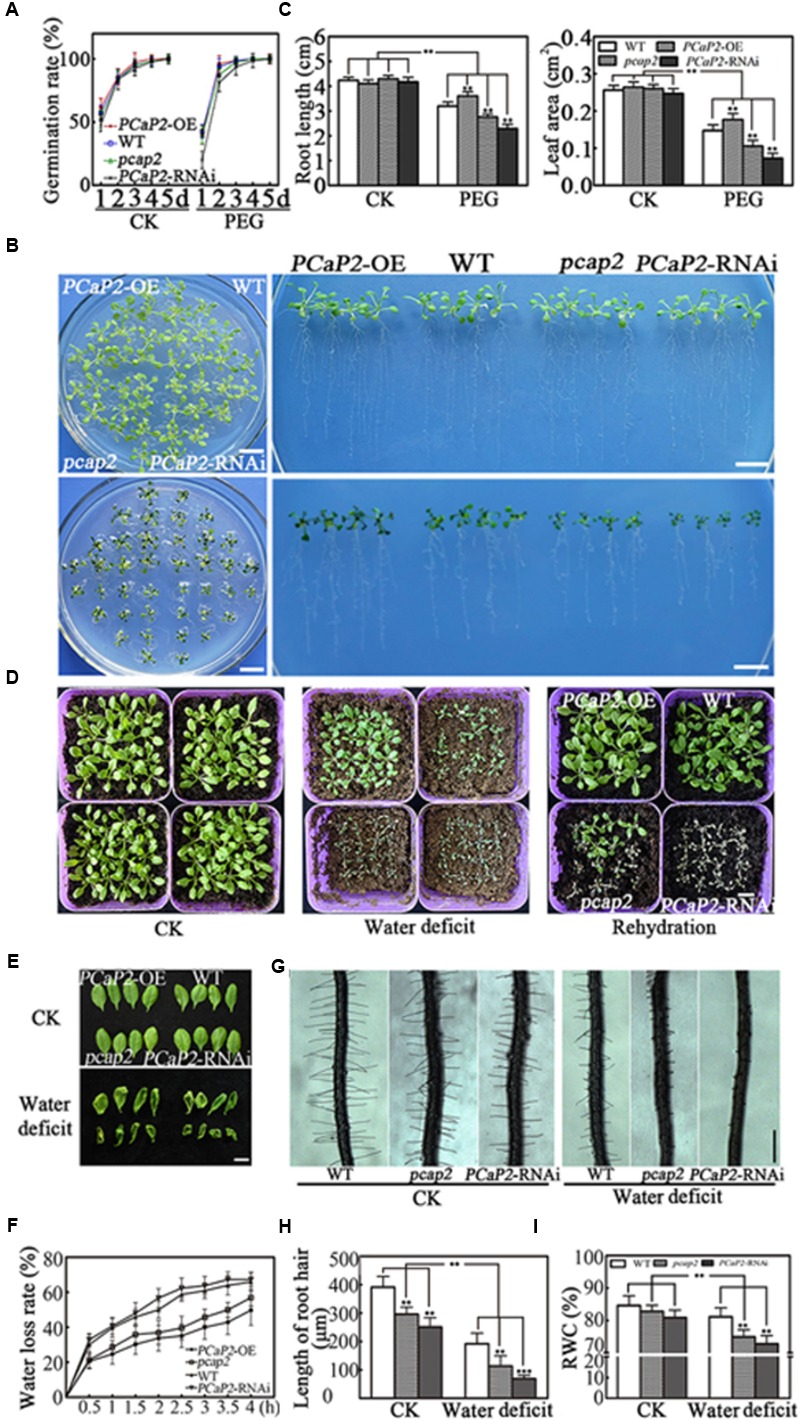
Effects of PCaP2 on Arabidopsis seed germination and seedling growth under water deficit stress. **(A)** Quantification of germination rates of the *PCaP2*-OE, WT, *pcap2* and *PCaP2*-RNAi seeds in 1/2 MS medium or coated with 25 % PEG from 1 to 5 d. At least 50 seeds from each sample were used for every technical replicate and three biological replicates were conducted. **(B)** Phenotypes of the *PCaP2*-OE, WT, *pcap2* and *PCaP2*-RNAi seedlings grown in water deficit. Scale bar = 1 cm. **(C)** The phenotypes statistics of the *PCaP2*-OE, WT, *pcap2* and *PCaP2*-RNAi seedlings grown in water deficit. At least 70 roots or 200 leaves from about 70 seedlings from each sample were used for every technical replicate and three biological replicates were conducted. **(D)** The survival status of *PCaP2*-OE, WT, *pcap2* and *PCaP2*-RNAi in normal, water deficit and rehydration matrix soil, respectively. Scale bar = 1 cm. **(E)** Phenotypes of leaves wilted of *PCaP2*-OE, WT, *pcap2* and *PCaP2*-RNAi plants for 0 h (up) and 4 h (down) under dehydration stress. Scale bar = 1 cm. **(F)** Water loss measurements for genotypes. Water loss in detached leaves was measured at the time points indicated. Water loss was expressed as a percentage of initial fresh weight. At least 30 detached leaves from each sample were used for every technical replicate and three biological replicates were conducted. **(G)** Phenotypes of root hairs in WT, *pcap2*, and *PCaP2*-RNAi lines in 1/2 MS or coated with 15% PEG. Scale bar = 0.5 mm. **(H)** The statistics of average root hair length in **(G)**. About 250 root hairs from 30 roots of each sample were used for every technical replicate and three biological replicates were conducted. **(I)** The relative water content (RWC) of WT, *pcap2* and *PCaP2*-RNAi lines in 1/2MS or coated with 15% PEG. About 30 seedlings from each sample were used for every technical replicate and three biological replicates were conducted. All data are mean values of three biological replicates ± SE. The significant difference was determined by ANOVA: ^∗^*P* < 0.05, ^∗∗^*P* < 0.01, ^∗∗∗^*P* < 0.001.

To elucidate the function of *PCaP2* in plant tolerance to water deficit, the previous identified one *PCaP2* overexpression (*PCaP2*-OE) line (Wang et al., 2007), one knockdown of T-DNA insertion (*pcap2*) line (SALK_021652; Kato et al., 2013; Zhu et al., 2013; Zhang et al., 2015; Kang et al., 2017), and one fully silenced *PCaP2* RNA interference (*PCaP2*-RNAi) line (Wang et al., 2007) were used. The *PCaP2* expression of these lines was analyzed by qRT-PCR which is consistent with the previous publications (Supplementary Figure S1). We firstly investigated their seed germination rates under normal or drought conditions. Under normal conditions, germination rates of *PCaP2*-RNAi and *pcap2* seeds were lower than those of *PCaP2*-OE and WT seeds at 1 day, and then these seeds gradually showed similar germination rate. At 4 and 5 days, the germination of these seeds was identical (**Figure 2A**). Water deficit significantly inhibited the germination of all seeds at 1 day, germination rates of *PCaP2*-RNAi were lower from 1 to 4 days, then the germination of these seeds was identical germination at 5 days (**Figure 2A**).

Next, the growth and survival status of the four-day-old seedlings were observed in water deficit treatments. It was found that the grown status showed no significant differences among WT, *PCaP2*-OE, *pcap2*, and *PCaP2*-RNAi lines under normal conditions. The *PCaP2*-OE seedlings showed longer roots and larger leaves than WT while *pcap2* and *PCaP2*-RNAi showed opposite growth phenotypes under water deficit (**Figures 2B,C**). The results of survival status after water deficit treatments showed that *PCaP2*-OE plants were more tolerant in response to water deficit than WT (**Figures 2C,D**) while *pcap2* and *PCaP2*-RNAi plants exhibited sensitivity to water deficit. To determine recovery after water deficit, we re-watered these seedlings for 7 days. The results showed that *PCaP2*-OE plants, WT, and some *pcap2* seedlings were recovered while all *PCaP2*-RNAi seedlings exhibited water deficit sensitivity (**Figure 2D**). The changes of leaf water loss and root water absorption in WT, *PCaP2*-OE, *pcap2*, and *PCaP2*-RNAi lines under water deficit conditions were observed. The results showed that *pcap2* and *PCaP2*-RNAi lines lost water faster and wilted earlier than WT in dehydration stress while *PCaP2*-OE seedlings showed opposite phenotypes (**Figures 2E,F**). Additionally, the *pcap2* and *PCaP2*-RNAi lines displayed shorter root hairs and less relative water content than WT in normal condition, the phenotypes were more significant in water deficit stress (**Figures 2G–I**). These results indicated that overexpression of *PCaP2* enhanced the tolerance of plants to water deficit and down of *PCaP2* led to plant water deficit hypersensitivity.

### The *PCaP2* Expression Is Highly Induced in Response to Exogenous ABA and SA Treatments

**FIGURE 3:**
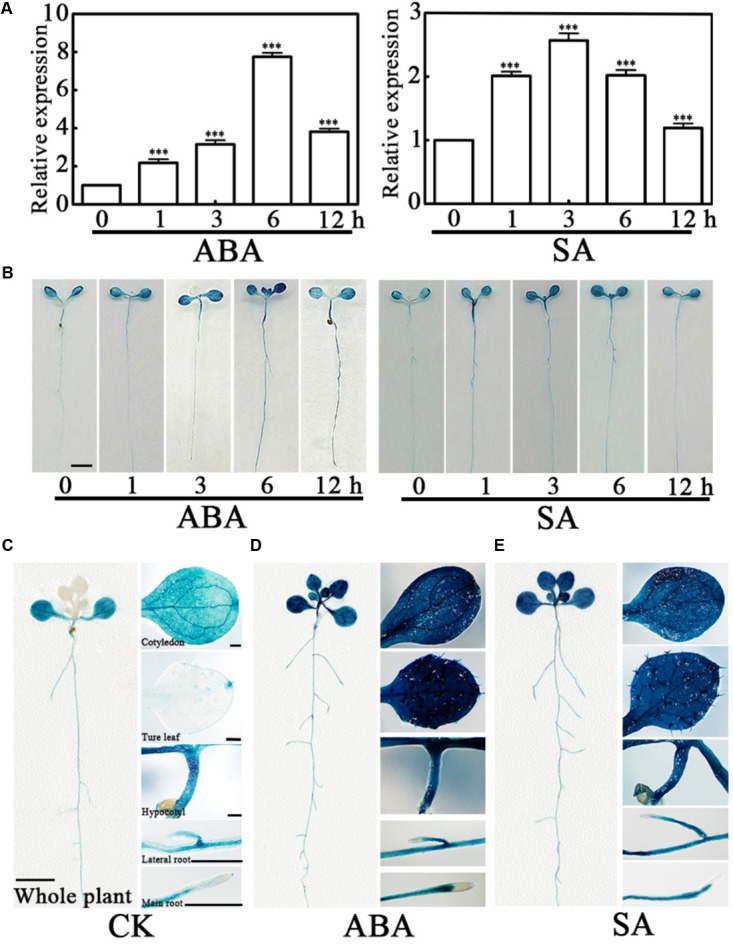
Expression pattern of *PCaP2* with exogenous ABA or SA treatments. **(A)** Relative expression of *PCaP2* in response to exogenous ABA and SA treatments. Fourteen-day-old seedlings were treated with 40 μM ABA or 100 μM SA for 0, 1, 3, 6, and 12 h by qRT-PCR analysis. Data represent mean values of three biological replicates ± SE. The significant difference was determined by ANOVA in comparison to 0 h: ^∗∗∗^*P* < 0.001. **(B)** Comparison of *PCaP2* expression levels with exogenous ABA or SA treatments by GUS staining. Seven-day-old seedlings of p*PCaP2*::GUS transgenic plants were treated with 40 μM ABA or 100 μM SA for 0, 1, 3, 6, and 12 h. Scale bar = 2.5 mm. **(C–E)** Analysis of the expression pattern of *PCaP2* by GUS staining of p*PCaP2*::GUS transgenic seedlings in normal (without ABA or SA treatments) **(C)** and 40 μM ABA treatments for 6 h **(D)** and 100 μM SA treatments for 6 h **(E)**. At least 15 seedlings from each sample were used for every technical replicate and three biological replicates were conducted. The scale bar is 5 mm in the pictures of whole plants, and 0.25 mm in the pictures of cotyledons, true leaves, hypocotyls, lateral roots, and main roots.

Abscisic acid and SA are important regulatory signals in water deficit stress, and the higher expression level of *PCaP2* has been found in exogenous 100 μM ABA and 100 μM SA treatments (Kato et al., 2010; Trivedi et al., 2016). To determine *PCaP2* in ABA and SA signaling pathways, the expression of *PCaP2* was examined in exogenous ABA and SA treatments by qRT-PCR and GUS staining assay in more details. Because the expression of many genes in Arabidopsis can be induced by 10–100 μM ABA (Zhu et al., 2007, 2017; Kato et al., 2010; Tian et al., 2015) and we found the expression of *PCaP2* is higher in 40 μM ABA treatments than that in 100 μM ABA treatment (**Figure 3A**; Kato et al., 2010). Thus, 40 μM ABA and 100 μM SA treatments were used in our studies. The results showed that the *PCaP2* mRNA level was significantly increased with ABA and SA treatments from 1 to 12 h, the peaks appeared at 6 h ABA and 3 h SA treatments (**Figures 3A,B**). The expression level of *PCaP2* was higher in 40 μM ABA than 100 μM SA treatments (**Figures 3A,B**). Further, the *PCaP2* expression was induced in the whole seedlings, cotyledons, true leaves, hypocotyls, primary roots, and lateral roots by ABA and SA treatments (**Figures 3B–E**), which were consistent with the results from water deficit treatments.

### *PCaP2*-OE Plants Show Increased Tolerance, While Its RNAi and Mutant Are More Sensitive in Exogenous ABA and SA Treatments

**FIGURE 4:**
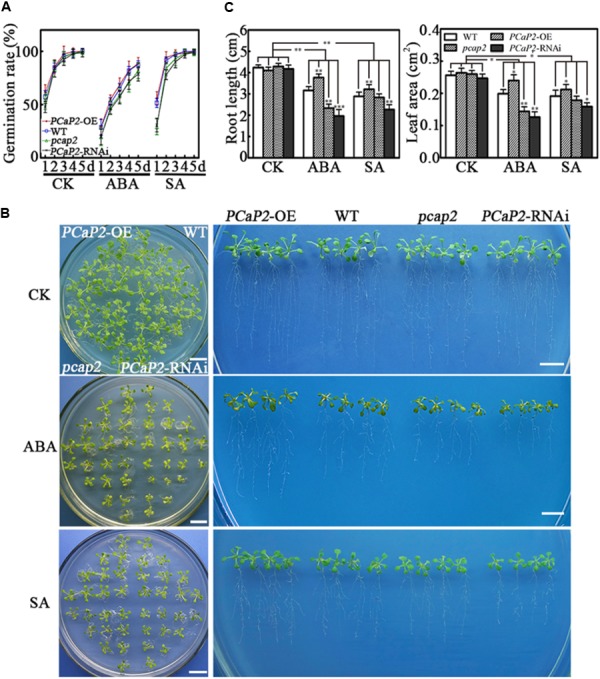
Effects of PCaP2 on Arabidopsis seed germination and seedling growth in ABA or SA treatments. **(A)** Quantification of germination rates of the *PCaP2*-OE, WT, *pcap2*, and *PCaP2*-RNAi seeds in 1/2 MS medium or mixed with 0.8 μM ABA and 0.3 mM SA from 1 to 5 days. A total of 40 seeds of each line were used for every technical replicate and three biological replicates were conducted. **(B)** Phenotypes of the *PCaP2*-OE, WT, *pcap2*, and *PCaP2*-RNAi seedlings grown in exogenous ABA and SA treatments. The seedlings were sown on 1/2 MS medium for 5 days, then transferred to 0.5 μM ABA or 0.05 mM SA medium and grew for 10 days. Scale bar = 1 cm. **(C)** The phenotype statistics of the *PCaP2*-OE, WT, *pcap2*, and *PCaP2*-RNAi seedlings grown in exogenous ABA and SA treatments. The main root length and leaf area of these seedlings were calculated after growth on 1/2 MS medium or supplemented with 0.5 μM ABA and 0.05 mM SA. At least 70 roots or 200 leaves from about 70 seedlings from each sample were used for every technical replicate and three biological replicates were conducted. All data are mean values of three biological replicates ± SE. The significant difference was determined by ANOVA: ^∗^*P* < 0.05, ^∗∗^*P* < 0.01, ^∗∗∗^*P* < 0.001.

Then we tested the germination rate of seeds and seedling growth of WT, *PCaP2*-OE, *pcap2*, and *PCaP2*-RNAi lines under normal, ABA, and SA treatments. To choose the most appropriate concentrations for ABA and SA treatments, 0.01–40 μM of ABA and SA were tested in the previous experiments used (data not shown). The results showed WT and *PCaP2*-OE showed the similar germination rates. The *PCaP2-*RNAi and *pcap2* showed much higher germination rates in 0.8 μM ABA and 0.3 mM SA treatments, compared to WT and *PCaP2*-OE (**Figure 4A**). The germination rates of *PCaP2*-RNAi and *pcap2* seeds were lower than those of *PCaP2*-OE and WT seeds in 1-to-5-day ABA and in 1-to-3-day SA treatments. The germination rates of these seeds were similar at day 4 of SA treatment and identical at day 5 of SA treatment. The growth status of WT, *PCaP2*-OE, *pcap2*, and *PCaP2*-RNAi lines was significantly different under 0.5 μM. ABA and 0.05 mM SA treatments (**Figures 4B,C**). The *PCaP2*-OE seedlings showed larger leaf area and longer primary roots than WT while *pcap2* and *PCaP2*-RNAi lines showed smaller leaves and shorter roots and root hairs, which were consistent with the water deficit inducible phenotypes (**Figures 4B,C**).

### PCaP2 Positively Regulates the Expression of *SnRK2* Genes and *PR* Genes Under Water Deficit Stress

To analyze whether the role of *PCaP2* in water deficit tolerance was mediated by ABA and SA signaling pathways, we firstly checked the *PCaP2* expression of WT, *PCaP2*-OE, *pcap2*, and *PCaP2*-RNAi in water deficit conditions by qRT-PCR. The analysis showed that the increasing expression of *PCaP2* in *PCaP2*-OE and the decreasing expression in *pcap2* and *PCaP2*-RNAi were compared with that in WT under water deficit treatments (Supplementary Figure S1).

**FIGURE 5:**
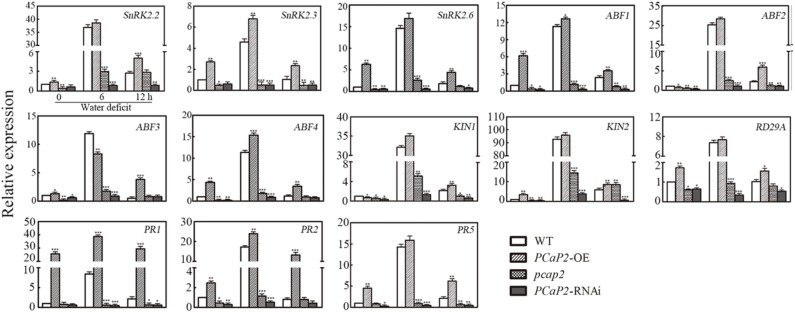
Effect of PCaP2 on the expression of ABA-, drought- and SA-responsive genes under water deficit. The relative gene expression of the 14-day-old seedlings of WT, *PCaP2*-OE, *pcap2*, and *PCaP2*-RNAi with water deficit treatments for 0, 6, and 12 h was determined by qRT-PCR analysis. Data represent mean values of three biological replicates ± SE. The significant difference was determined by ANOVA: ^∗^*P* < 0.05, ^∗∗^*P* < 0.01, ^∗∗∗^*P* < 0.001.

Next, we examined the expression of the following ABA-responsive genes, including *SnRK2.2*, -*2.3*, -*2.6*, *ABF2*, *-3*, -*4* (Choi et al., 2000; Uno et al., 2000; Boudsocq et al., 2004; Gong et al., 2015; Jia et al., 2015); drought-inducible genes, including *RD29A* (Yamaguchi-Shinozaki and Shinozaki, 1994); *KIN1* and *KIN2* (Kurkela and Borg-Franck, 1992); and SA-responsive genes such as *PR1*, *-2*, *-5* genes (Liu P. et al., 2013; Liu W.X. et al., 2013) in WT, *PCaP2*-OE, *pcap2* and *PCaP2*-RNAi with dehydration conditions at 0, 6, and 12 h by qRT-PCR analysis. Under normal conditions, *pcap2* and *PCaP2*-RNAi inhibited the expression of ABA- and drought-responsive genes, except SA-responsive genes (**Figure 5**), and *PCaP2*-OE increased the expression of SA-responsive genes, suggesting that PCaP2 might be the complex mechanisms in regulating gene expression in normal conditions. Under water deficit stress, the expression of all these genes, including ABA-, drought-, and SA-responsive genes, was significantly inhibited in *pcap2* and *PCaP2*-RNAi while increased in *PCaP2*-OE, compared with that in WT, indicating that PCaP2 was involved in regulating drought-, ABA- and SA-responsive genes under water deficit stress.

### PCaP2 Positively Regulates ABA-Mediated *SnRK2*s Expression and SA-Mediated *PR*s Expression in Water Deficit

**FIGURE 6:**
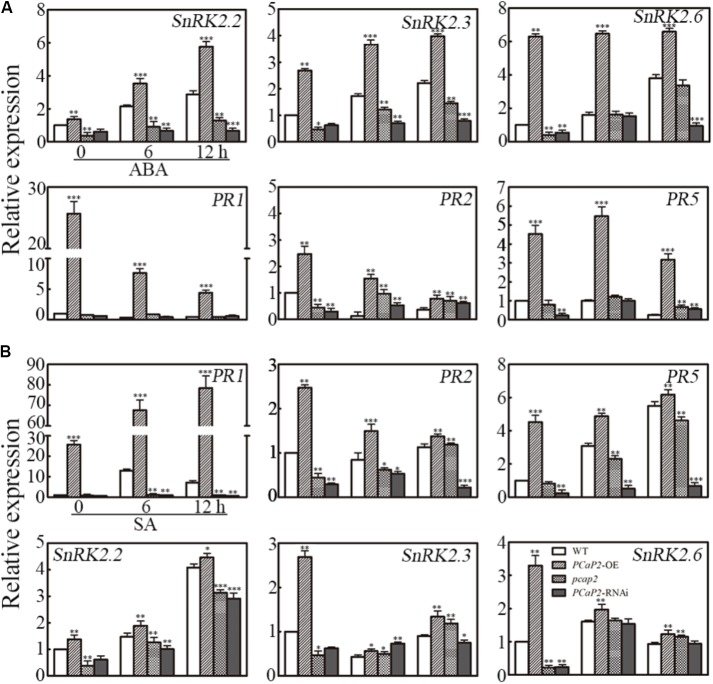
Effect of PCaP2 on the expression of *SnRK2* and *PR* genes with exogenous ABA and SA. **(A)** The relative expression of *SnRK2* genes and *PR* genes of the 14-day-old plants with 40 μM ABA treatments for 0, 6, and 12 h was determined in WT, *PCaP2*-OE, *pcap2*, and *PCaP2*-RNAi by qRT-PCR assay. **(B)** The relative expression of *PR* and *SnRK2* genes of the 14-day-old plants with 100 μM SA treatments for 0, 6, and 12 h was determined in WT, *PCaP2*-OE, *pcap2*, and *PCaP2*-RNAi by qRT-PCR assay. Data represent mean values of three biological replicates ± SE. The significant difference was determined by ANOVA: ^∗^*P* < 0.05, ^∗∗^*P* < 0.01, ^∗∗∗^*P* < 0.001.

To further analyze whether the effect PCaP*2* on gene expression in response to water deficit was mediated by ABA and SA signaling pathways, we firstly checked the *PCaP2* expression of WT, *PCaP2*-OE, *pcap2* and *PCaP2*-RNAi in ABA and SA treatments by qRT-PCR. The analysis showed that the increase expression of *PCaP2* in *PCaP2*-OE and the decreasing expression in *pcap2* and *PCaP2*-RNAi were compared with that in WT under ABA and SA treatments (Supplementary Figure S1). Next, we examined the expression of *SnRK2.2*, -*2.3*, -*2.6* and *PR1*, *-2*, *-5* genes in WT, *PCaP2*-OE, *pcap2*, and *PCaP2*-RNAi with ABA and SA treatments at 0, 6, and 12 h by qRT-PCR analysis. The results showed that the effect of *PCaP2*-OE, *pcap2*, and *PCaP2*-RNAi on affecting the ABA-responsive gene expression in ABA treatments, rather than SA treatments, were similar to that in drought treatments, (**Figure 6A**). In contrast, the effect of *PCaP2*-OE, *pcap2*, and *PCaP2*-RNAi on affecting the SA-responsive gene expression in SA treatments, rather than ABA treatments, were similar to that in drought treatments (**Figure 6B**). These results illustrated that PCaP2 regulated the expression of ABA-mediated *SnRK2* genes and SA-mediated *PR* genes under water deficit stress, indicating that PCaP2 was a crosstalk factor in response to ABA and SA signals in water deficit.

## Discussion

Water deficit significantly influences plant growth, development, and productivity. One of the most important regulators in drought is ABA, and some cases of SA-mediated plant water deficit tolerance have been documented. However, details of crosstalk between ABA and SA signaling cascades in response to water deficit remain largely unknown. Previous studies have suggested that PCaP2 responds to some phytohormone signaling pathways and abiotic stress through mRNA expression pattern analysis (Kato et al., 2010). Our results further support a key role for PCaP2 in Arabidopsis water deficit tolerance and suggest that it is connected to the main ABA and SA signaling pathways.

Previous studies have shown that *PCaP2* is mainly expressed in roots and flower tissues in normal conditions, and its mRNA level in whole seedlings is induced by drought as well as treatment with ABA or SA (Wang et al., 2007; Kato et al., 2010). Compared with previous studies, our data provide more detailed findings. High expression of *PCaP2* in roots was found under normal and stress conditions. This might be related to its function in root water absorption and root hair growth in both normal and water deficit conditions. Additionally, *PCaP2* was not expressed in true leaves under normal conditions, but was highly induced in water deficit and treatment with ABA or SA. This indicated that in true leaves, the increase of PCaP2 level is a key response to stress that is triggered by phytohormone signals, which may be associated with its function in controlling water deficit-induced leaf water loss.

**FIGURE 7:**
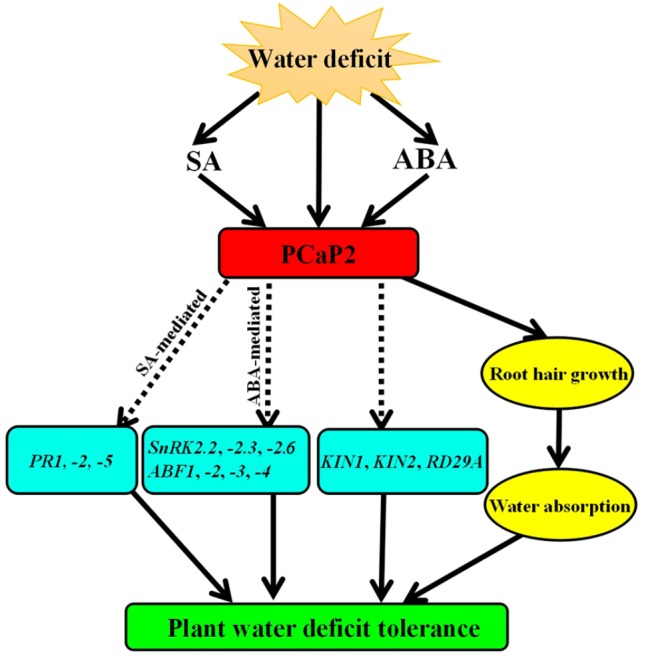
Working model of PCaP2 function in plant response to water deficit stress. Arrows ending solid lines indicate positive and direct regulation, arrows ending broken lines denote hypothetical or indirect regulation. Details of this model are discussed in the text.

Some ABA-sensitive seedlings show improved drought tolerance in soil, such as *SnRK2s*-OE and *AREBs*-OE, while some mutants show the same sensitivity to ABA and drought treatments, such as *Atrgga* and *AtDi19-3* (Fujita et al., 2009; Qin et al., 2014; Ambrosone et al., 2015). Generally, ABA-sensitive mutants improve drought tolerance by regulating stomatal movement. However, changes in the drought tolerance of *Atrgga* and *AtDi19-3* are not dependent on stomatal movement (Fujita et al., 2009; Qin et al., 2014; Ambrosone et al., 2015). In the present study, we used the previously identified *PCaP2*-OE line (Wang et al., 2007) *pcap2* mutant (Kato et al., 2013; Zhu et al., 2013; Zhang et al., 2015; Kang et al., 2017) and *PCaP2*-RNAi lines (Wang et al., 2007), and our analysis of *PCaP2* expression of these lines is the same with the prior findings. Our results showed that *pcap2* and *PCaP2*-RNAi lines were more sensitive to ABA and drought, while *PCaP2*-OE plants were more resistant to ABA and drought stress. Although our results do not address whether PCaP2 can regulate stomatal movement, it is possible that PCaP2 has a similar mechanism to AtRGGA and AtDi19-3. PCaP2 do not fully depend on the regulation of stomatal movement in response to drought stress; PCaP2 functionality in plant water deficit tolerance can be borne out by regulating root hair growth to benefit water absorption and to induce the expression of numerous key drought-responsive genes (**Figure 7**). Furthermore, the water deficit-inducible phenotypes in fully silenced *PCaP2*-RNAi plants were greater in magnitude to those in knockdown *pcap2* mutants. This suggests that the role of PCaP2 in water deficit is dependent on its inducible expression level. These results indicate that PCaP2 is a positive regulator of water deficit tolerance in Arabidopsis.

Root hairs are the major water absorbing tissue in plants. Under water-limited conditions, root hair growth is significantly inhibited, which affects root water absorption and plant water deficit tolerance (Worrall and Roughley, 1976; Zahran and Sprent, 1986; Schnall and Quatrano, 1992). Our results showed that reduced expression of *PCaP2* leads to shorter root hairs under normal conditions, which is consistent with previous results from Zhang et al. (2015). Moreover, significant inhibition of shorter root hair development was seen under water deficit conditions in *pcap2* and *PCaP2*-RNAi, which could be one main pathway through which PCaP2 induces plant water deficit tolerance.

In Arabidopsis, *SnRK2.2*, *-2.3*, and *-2.6* are crucial in the response to water deficit stress (Shi and Yang, 2014; Trivedi et al., 2016). Prior studies have shown that *SnRK2.2, -2.3*, and *-2.6* triple mutants are insensitive to ABA and water deficit treatments (Fujii and Zhu, 2009; Fujita et al., 2009). Thus, PCaP2 mainly influences plant water deficit tolerance by regulating the ABA-mediated SnRK2 signaling pathway. It has been shown that *SnRK2.6* functionally separates from *SnRK2.2* and *-2.3* under water deficit stress (Fujii et al., 2007; Kulik et al., 2011). Furthermore, *SnRK2.2* and *SnRK2.3* kinases regulate seed dormancy, germination, and seedling growth under water deficit conditions (Fujii et al., 2007; Fujita et al., 2009). SnRK2.6/OST1 plays an important role in stomatal movement (Vlad et al., 2009; Vahisalu et al., 2010; Brandt et al., 2012; Acharya et al., 2013; Imes et al., 2013; Grondin et al., 2015; Matrosova et al., 2015; Yin et al., 2016), suggesting that it is involved in plant drought tolerance by regulating stomatal movement. Some studies have further demonstrated that *SnRK2.2*, *-2.3*, and *-2.6* are crucial in the response of Arabidopsis to ABA and water deficit stress (Shi and Yang, 2014; Trivedi et al., 2016). The expression of ABA-dependent transcription factors, such as ABIs and ABFs, is also stimulated by SnRK2s (Fujii et al., 2007). These factors mediate the expression of downstream ABA-inducible genes to improve plant water deficit resistance. Our results illustrated that *PCaP2* increased *SnRK2.2*, -*2.3*, and -*2.6* expression as well as *SnRK2*-mediated gene expression, such as *ABF1*, *-2*, *-3*, *-4*, *KIN1*, *KIN2*, and *RD29*, in water deficit stress and ABA treatments. Consequently, it can be speculated that water deficit stress-induced PCaP2 may affect the ABA-mediated SnRK2 signaling pathway and thus regulate drought-inducible gene expression, seed germination, seedling growth, and leaf water loss.

Compared to the ABA signaling pathway, relatively few cases of SA-mediated drought tolerance have been presented, Kang G. et al. (2012) and Kang G.Z. et al. (2012) identified 76 proteins as potentially involved in the SA signaling pathway in drought-exposed *T. aestivum*. Genetic and gene expression analysis of these proteins show that *PR1*, *-2*, and *-5* are required in SA signaling in drought stress (Liu P. et al., 2013; Liu W.X. et al., 2013). Arabidopsis *cpr5* and *acd6* mutants exhibit SA accumulation, and their drought tolerance is improved by inducing SA-mediated expression of *PR1*, -*2*, and -*5* (Liu P. et al., 2013). Additionally, *PR1*, -*2*, and -*5* overexpression enhances drought tolerance in Arabidopsis (Liu W.X. et al., 2013; Qin et al., 2014).

Transcription factor Di19 regulates the expression of *PR1*, -*2*, and -*5* in drought conditions (Liu W.X. et al., 2013; Qin et al., 2014). Water loss rates of *PR1*-, *PR2*-, and *PR5*-overexpressing lines are lower than those of WT plants, and the three genes are highly expressed in stomata, suggesting that PR1, PR2, and PR5 may regulate stomatal movement in response to drought tolerance (Liu W.X. et al., 2013; Qin et al., 2014). Additionally, PRs play significant roles in regulating phytohormone-signaling, such as auxin and JA (Thaler et al., 1999; Wang et al., 2001; Wen et al., 2008; Iglesias et al., 2011), suggesting that PR1, PR2, and PR5 may mediate the complex crosstalk between phytohormone signals under drought stress. However, complete mechanisms of PR actions under drought stress are largely unknown. Our results show that *PCaP2* positively regulates the expression of *PR1*, *-2*, and *-5* after SA and water deficit treatments, but not ABA treatment. In addition, *PCaP2*-RNAi and *pcap2* lines lost water faster and wilted earlier than WT lines, which stands in contrast to the phenotypes of *PR1*-, *PR2*-, and *PR5*-overexpressing seedlings (Liu W.X. et al., 2013; Qin et al., 2014). However, the phenotypes of *PCaP2*-OE plants are consistent with those of *PRs*-overexpressing seedlings. Thus, these results support the idea that *PCaP2* activates the key SA-mediated signaling pathway in response to water deficit, providing a new pathway to regulate *PR*s under water deficit conditions.

Interactions between SA and ABA signals have been shown to occur during abiotic stress, such as salt and cold stress. In *S. lycopersicum*, treatment with SA improves plant growth, osmotic adaptation, and ABA accumulation under normal conditions and during salt stress (Szepesi et al., 2009). Under cold conditions, exogenous ABA treatment increases endogenous SA level and oHCA content in *Z. mays*, suggesting that the ABA signal may combine with SA-related stress responses during cold stress (Szalai et al., 2011). Our results showed that ABA and SA can trigger *PCaP2*, and that *PCaP2* then regulates the ABA-mediated SnRK2 signaling pathway as well as the expression of SA-mediated *PR* genes during water deficit stress. Thus, PCaP2 mediates crosstalk in response to ABA and SA signals during water deficit.

We have not interrogated whether *PCaP2* can directly regulate *SnRK2*s and/or *PR*s in normal, water deficit, or exogenous ABA and SA conditions. However, previous studies have shown that PCaP2 can bind PtdInsPs, Ca^2+^ and Ca^2+^/CaM complexes. These ligands are important components of intracellular signaling networks involved in plant water deficit tolerance (Zielinski, 1998; Meijer and Munnik, 2003; Carlton and Cullen, 2005; Perera et al., 2008; DeFalco et al., 2009; Kleerekoper and Putkey, 2009; Luan, 2009; Xue et al., 2009; Kato et al., 2010, 2013). Our results also showed that PCaP2 regulated the expression of many key ABA-responsive genes, SA-responsive genes, and drought-inducible genes, including upstream genes such as *SnRK2.2*, -*2.3*, and -*2.6*. Thus, it is well-accepted that *PCaP2* mainly functions as a Ca^2+^-binding protein to participate in intracellular signaling transduction under water deficit stress.

In conclusion, water deficit triggers ABA and SA accumulation, which, in turn, induce *PCaP2* expression. PCaP2 then activates the expression of many key drought-responsive genes, including ABA-mediated genes (*SnRK2.2*, *-2.3*, *-2.6*, *ABF1*, *-2*, *-3*, *-4*) and SA-mediated genes (*PR1*, *-2*, *-5*) and drought-inducible genes (*RD29A*, *KIN1* and *KIN2*), and affects root hair growth to increase water absorption, which improve plant water deficit tolerance (**Figure 7**). This study highlights the positive and important role that PCaP2 plays in plant water deficit tolerance by mediating crosstalk between ABA and SA signals, providing novel evidence relating to the underlying and complex mechanisms mediated by ABA and SA signals during plant water deficit tolerance.

## Materials and Methods

### Plant Materials, Growth Conditions, and Treatments

Arabidopsis ecotype Col-0 was the background for all transgenic and mutant plants in this study. The seeds of *PCaP2*-OE line (Wang et al., 2007), *pcap2* mutant (Kato et al., 2013; Zhu et al., 2013; Zhang et al., 2015; Kang et al., 2017), and *PCaP2*-RNAi line (Wang et al., 2007) and *PCaP2* promoter::GUS (p*PCaP2::GUS*) (Wang et al., 2007) were provided by Professor Ming Yuan, China Agricultural University, Beijing, China. All these seeds were used in the previous reports.

Surface sterilized Arabidopsis seeds were sown on MS plates with 1/2 MS (Murashige & Skoog) medium (pH adjusted to 5.8–6.0 with 1 M NaOH) with vitamins, 0.6 % phytoagar (PlantMedia), and 1% sucrose, and then in darkness at 4°C for 3 days. Seedlings were then transferred to a growth chamber at 22°C, with a 16 h/8 h (light/dark) photoperiod at approximately 120 μmol m^-2^ s^-1^. The 1/2 MS plus 1% sucrose media are usually used in water deficit, ABA, and SA treatments (Jayakannan et al., 2015; Schwalm et al., 2017; Zhu et al., 2017). *PCaP*2-OE, WT, *pcap2*, and PC*aP2*-RNAi were used for all experiments. For germination rate analysis, seeds were sown on 1/2 MS medium with 25 % PEG (dehydration), 0.8 μM ABA and 0.3 mM SA for 1, 2, 3, 4, 5 days, respectively. For seedling growth experiments, 4-day-old seedlings were transferred to soil for 7 days, then with 40 % PEG (dehydration), 0.5 μM ABA and 0.05 mM SA for 10 days. For survival experiments, 4-day-old seedlings were transferred to soil for 7 days, then were treated for 10 days without water, following with water for 7 days. At least 20 seedlings were harvested for observing in three biological replicates in each experiment. The best concentration of ABA and SA in the present paper was chosen by the prepared experiments for 0.01 to 40 μM of ABA and SA (data not shown), because the previous findings show that significantly different ABA and SA concentrations from 1 nM to 80 μM are used in seed germination and seedling growth experiments (Reyes and Chua, 2007; Zhu et al., 2007; Horváth et al., 2015; Huang et al., 2017). For water-loss assays, rosette leaves of comparable size from 4-week-old plants grown under long days were detached, placed on a Petri dish and weighted at a specified time (0, 0.5, 1, 1.5, 2, 2.5, 3, 3.5, and 4 h) after detachment. For root hair growth assays, 3-day-old seedlings were transferred to 1/2 MS medium with or without 15% PEG for 3 days, and the root hair length was assayed by ImageJ software^1^. Three-day-old seedlings transferred to 1/2 MS medium with or without 15% PEG for 7 days were used to test relative water content. Relative water content was measured as previous reports described (Brini et al., 2007).

### RNA Isolation and Quantitative RT-PCR (qRT-PCR) Analysis

Total RNA was isolated from 1-day-old seedlings using RNasy plant mini kit (Qiagen), treated with RNase-Free DNase (Qiagen) at 37°C for 1 h to degrade genomic DNA, and 1 μg total RNA to synthesize cDNA by oligo-(dT) 20-primed reverse transcription by the Omniscript RT Kit (Qiagen). The cDNA was amplified using SuperReal PreMix Plus (SYBR Green, TIANGEN, China) in a 10 μL volume. The expression levels of *18S* rRNA was used as an internal control. Analysis was performed using the BioRad Real-Time System CFX96TM C1000 Thermal Cycler (Singapore).

For the expression of *PCaP2* under drought, ABA, and SA treatment assays, 14-day-old seedlings from WT plants were exposed 1/2 MS medium with or without drought, 40 μM ABA and 100 μM SA for 0, 1, 3, 6, 9, and 12 h. The previous finding showed the higher expression level of *PCaP2* has been found in exogenous 100 μM ABA and 100 μM SA treatments (Kato et al., 2010). The expression of many genes can be increased in 10 to 100 μM ABA treatments in Arabidopsis (Zhu et al., 2007; Kato et al., 2010; Tian et al., 2015; Zhu et al., 2017). We found the expression level of *PCaP2* can be induced in 40 μM ABA treatments, and the expression is higher than that in 100 μM ABA treatments (**Figures 3A**; Kato et al., 2010). Thus 40 μM ABA was used in our experiments. At each time point, all seedlings were immediately frozen by liquid nitrogen and then stored at -80°C for RNA preparation. To assay the expression of drought-, ABA- and SA-responsive genes under water deficit stress in WT and *pcap2*, WT and mutant seedlings grown at normal conditions for 14 days were harvested and treated by dehydration stress for 0, 6, 12 h. To assay the expression of *SnRK2* and *PR* genes in WT and *pcap2* treatment with ABA and SA, the seedlings of 14-day-old at normal condition were harvested and treated with 40 μM ABA and 100 μM SA for 0, 6, 12 h. Total RNA extraction and reverse transcription were performed as described above. All primer pairs used for qRT-PCR examination are listed in Supplementary Table S1. Each representative experiment was performed with at least three replicates.

### Histochemical Staining of GUS Activity

Seven-day-old or 14-day-old p*PCaP2::GUS* seedlings treated with 40% PEG, 40 μM ABA, or 100 μM SA for 0, 1, 3, 6, and 12 h were collected for observing the changes of GUS activity. The GUS staining procedure was executed by the method mentioned in Wang et al. (2007). Samples were observed on an Olympus microscope equipped with a color CCD camera (Sutter Instrument; LAMBDA 10-2) or by an Epson scanner.

### Statistical Analysis

The experiments were conducted at least three times, each of which contained three technical replicates. Data presented as the mean ± SE of three biological replicates. The significant difference was analyzed by SPSS statistical software (ver.16.0, SPSS Inc., Chicago, IL, United States) via one-way or two-way ANOVA (^∗^*P* < 0.05, ^∗∗^*P* < 0.01, ^∗∗∗^*P* < 0.001). In addition, we tested the normality of data about the main root length, leaf area, and root hair length under drought stress with Lilliefors corrected K-S^a^ test and Shapiro–Wilk test by SPSS (Supplementary Table S2). The detailed information of the chemicals and kits used in this study are listed in Supplementary Table S3.

## Author Contributions

CW and XW designed the study. YW, LW, HL, BZ, and QC performed the experiments and data analysis. XL, SB, YL, and QW provided help in experimental methods. SZ participated in the discussion. MH and ST trained the use of experimental equipment. SY helped to revised the language and grammar. CW wrote the manuscript.

## Conflict of Interest Statement

The authors declare that the research was conducted in the absence of any commercial or financial relationships that could be construed as a potential conflict of interest.

## Footnotes

1 http://rsb.info.nih.gov/ij/

## Abbreviations

ABA: abscisic acid
ABF: abscisic acid responsive element-binding factor
ABI: abscisic acid insensitive
AREBs/ABFs: ABRE-binding proteins/factors
Ca^2+^: calcium ion
CaM: calmodulin
CCD: charge-coupled device
CPK: calcium-dependent protein kinase
Di19: drought-induced 19
GA: gibberellin
GR: glutathione reductase
GST: glutathione s-transferase
GUS: β-glucuronidase
IAA: indole-3-acetic acid
JA: jasmonic acid
KIN: kinase
MDHAR: Monodehydroascorbate reductase
MAP18/PCaP2: microtubule-associated protein18/plasma membrane-associated Ca^2+^-binding protein-2
MT: microtubule
MS: Murashige Skoog
NO: nitric oxide
OST1: open stomata1
PA: phosphatidic acid
*PCaP2*-OE: *PCaP2* overexpression
*PCaP2*-RNAi: *PCaP2* RNA interference
PEG: polyethylene glycol
PR: pathogenesis-related
PtdInsPs: phosphatidylinositol phosphates
qRT-PCR: quantitative real-time PCR
RD29A: responsive to desiccation 29A
RT-PCR: reverse transcriptase-mediated PCR
SA: salicylic acid
*S. lycopersicum*: Solanum lycopersicum
SNF: sucrose non-fermenting
SnRK2: SNF1-related protein kinase 2
WT: wild type
*Z. mays*: Zea mays
RGGA: Arginine Glycine Glycine (RGG) box-containing RNA-binding protein
Di19: drought-induced protein19.
