# Corrigendum: Arabidopsis PCaP2 Functions as a Linker Between ABA and SA Signals in Plant Water Deficit Tolerance

**DOI:** 10.3389/fpls.2018.01062

**Published:** 2018-07-25

**Authors:** Xianling Wang, Yu Wang, Lu Wang, Huan Liu, Bing Zhang, Qijiang Cao, Xinyu Liu, Shuangtian Bi, Yanling Lv, Qiuyang Wang, Shaobin Zhang, Ming He, Shuang Tang, Shuo Yao, Che Wang

**Affiliations:** ^1^College of Biological Science and Biotechnology, Shenyang Agricultural University, Shenyang, China; ^2^Department of Medicine, HE University School of Clinical Medicine, Shenyang, China; ^3^Vegetable Research Institute of Liaoning Academy of Agricultural Sciences, Shenyang, China

**Keywords:** PCaP2, water deficit, ABA, SA, SnRK2, PR, Arabidopsis

In the original article, there was a mistake in the legend for **Figure 4** as published. The concentration of ABA was wrong, it should be 0.5 μM but not 0.5 mM. The correct legend appears below.

**Figure 4** Effects of PCaP2 on Arabidopsis seed germination and seedling growth in ABA or SA treatments. **(A)** Quantification of germination rates of the *PCaP2*-OE, WT, *pcap2* and *PCaP2*-RNAi seeds in 1/2 MS medium or mixed with 0.8 μM ABA and 0.3 mM SA from 1 to 5 d. A total of 40 seeds of each line were used for every technical replicate and three biological replicates were conducted. **(B)** Phenotypes of the *PCaP2*-OE, WT, *pcap2* and *PCaP2*-RNAi seedlings grown in exogenous ABA and SA treatments. The seedlings were sown on 1/2 MS medium for 5 d, then transferred to 0.5 μM ABA or 0.05 mM SA medium and grew for 10 d. Scale bar = 1 cm. **(C)** The phenotype statistics of the *PCaP2*-OE, WT, *pcap2* and *PCaP2*-RNAi seedlings grown in exogenous ABA and SA treatments. The main root length and leaf area of these seedlings were calculated after growth on 1/2 MS medium or supplemented with 0.5 μM ABA and 0.05 mM SA. At least 70 roots or 200 leaves from about 70 seedlings from each sample were used for every technical replicate and three biological replicates were conducted. All data are mean values of three biological replicates ± SE. The significant difference was determined by ANOVA: ^*^P < 0.05, ^**^P < 0.01, ^***^P < 0.001.

In addition, Qiuyang Wang was not included as an author in the published article. The updated author contributions statement appears below.

## Author contributions

CW and XW designed the study. YW, LW, HL, BZ, and QC performed the experiments and data analysis. XL, SB, YL, and QW provided help in experimental methods. SZ participated in the discussion. MH and ST trained the use of experimental equipment. SY helped to revised the language and grammar. CW wrote the manuscript.

The authors apologize for these errors and state that this does not change the scientific conclusions of the article in any way.

The original article has been updated.

### Conflict of interest statement

The authors declare that the research was conducted in the absence of any commercial or financial relationships that could be construed as a potential conflict of interest.

