# Supplementary figures and images for: Arabidopsis PCaP2 Functions as a Linker Between ABA and SA Signals in Plant Water Deficit Tolerance
Source: Front Plant Sci. 2018 May 8;9:578. doi: 10.3389/fpls.2018.00578 (PMC5962825; doi:10.3389/fpls.2018.00578)

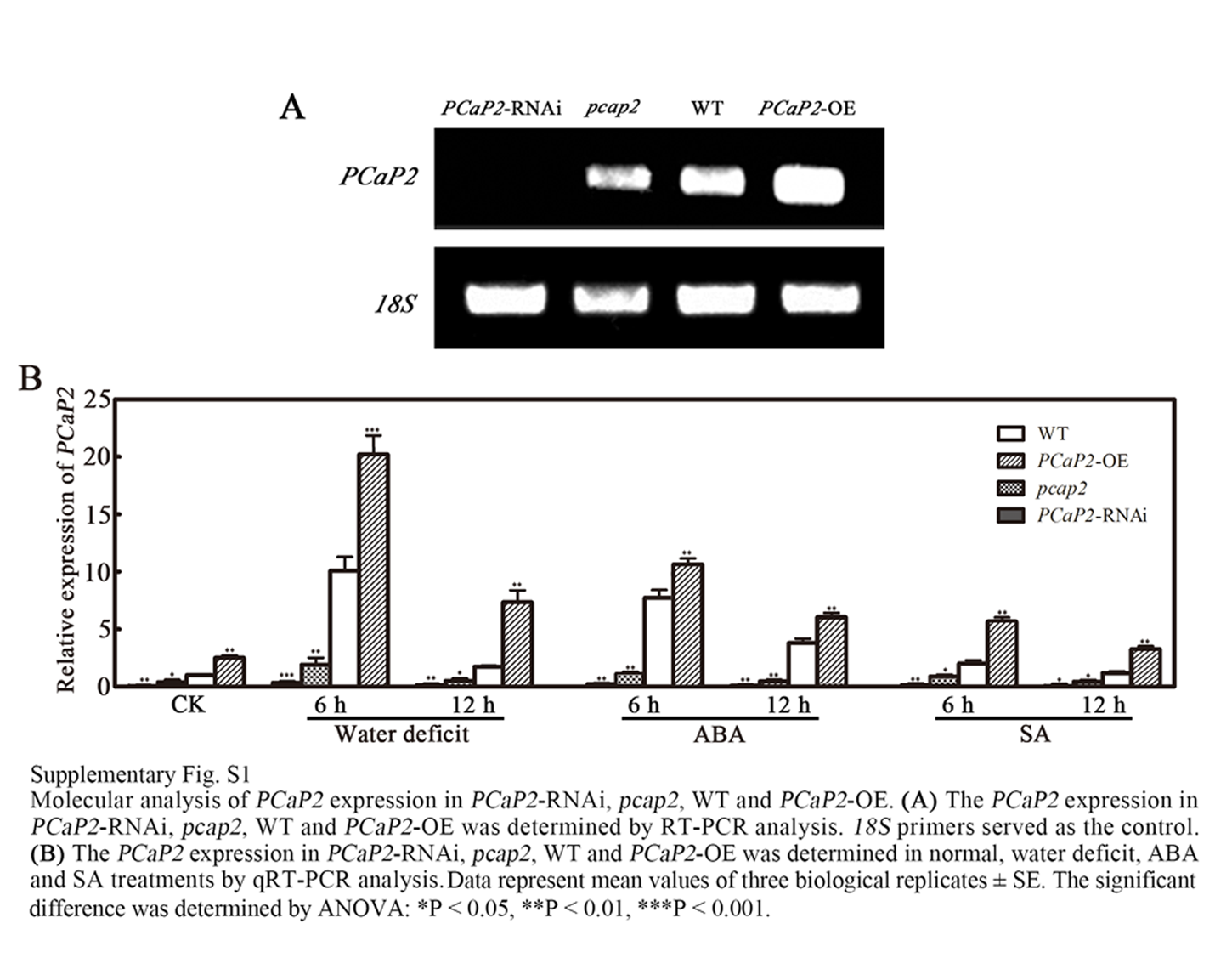

Supplement: Supplementary file 1 [file Image_1.tif]
