# Supplementary material for: Arabidopsis PCaP2 Functions as a Linker Between ABA and SA Signals in Plant Water Deficit Tolerance
Source: Front Plant Sci. 2018 May 8;9:578. doi: 10.3389/fpls.2018.00578 (PMC5962825; doi:10.3389/fpls.2018.00578)
Supplement: Supplementary file 2 [file Table_1.docx]

**Supplementary Table S1** Primers used in this study

| Gene name | Primer sequence (5’ to 3’) |
| --- | --- |
| *SnRK2.2* | Forward: TGGAATATGCTGCTGGTGGAGAACTTTATGAG |
|  | Reverse: TGGTTGGGAATGAAGAACAGAAGACTTGAGAG |
| *SnRK2.3* | Forward: TCTGGCGGTGAACTTTACGAGCGGATTTG |
|  | Reverse: CCTGACGAAGCAGTACCTCTGGAGCGATG |
| *SnRK2.6* | Forward: CACAGAGATTGAAGTCGCAAAGAGACAGAG |
|  | Reverse: CGGAGCCAATATCCTTGACGAGTTCATACC |
| *ABF1* | Forward: GGAGAAGGTTGTTGAGAGAAGG |
|  | Reverse: AGCTTCCAGTTCCAAGGTATAAG |
| *ABF2* | Forward: CACAGCACCAACGCCTAAAGC |
|  | Reverse: ATCGCAACAGCAACAGCCAATC |
| *ABF3* | Forward: ATCGTCCGAGGCAAGGTAAGTG |
|  | Reverse: TGATGGTGTGAGTGAGCAGCAG |
| *ABF4* | Forward: ATCTGCTGTTGTTGCTGCTGAAG |
|  | Reverse: ACTGCTGCTGGCGGCTTAG |
| *KIN1* | Forward: GGAAGGCATTCTTGTTGGTCTCTG |
|  | Reverse: GCCCACATCTCTTCTCATCATCAC |
| *KIN2* | Forward: ACTCGGATCGCTACTTGTTCAGG |
|  | Reverse: AGGCGGGAAAGAGTATATCGGATG |
| *RD29A* | Forward: GTCTCCGTCTTTGGGTCTCTTCC |
|  | Reverse: TTCTCCGATGGGCTTTGGTAGTG |
| *PR1* | Forward: CTACGCAGAACAACTAAGAGGCAAC |
|  | Reverse: CCTTCTCGCTAACCCACAT |
| *PR2* | Forward: GCTCGTGAATCTCTACCCTTAC |
|  | Reverse: GTATGAGTACCCTGGATCGTTATC |
| *PR5* | Forward: GAGGATCGGGAGATTGCAAATA |
|  | Reverse: ATTAAACCTCTCACAGGCACTC |
| *18s rRNA* | Forward: CGGCTACCACATCCAAGGAA |
|  | Reverse: GCTGGAATTACCGCGGCT |
| *PCaP2* | Forward: CCAGCCGTAGAAGAGGAGAAGAAG |
|  | Reverse: GGAGTTTCGGGAGCCTTAGTCG |

**Supplementary Table S2**

Normality test for main root length, leaf area and root hair length of *PCaP2*-OE, WT, *pcap2* and *PCaP2*-RNAi lines grown in drought stress provided by SPSS.

| Samples | | Kolmogorov-Smirnov^a^ With Lilliefors Correction Test | | | Shapiro-Wilk Test | | |
| --- | --- | --- | --- | --- | --- | --- | --- |
|  |  | Statistics | Df | *p*-value | Statistics | Df | *p*-value |
|  | | Main root length | | | | | |
| Control | *PCaP2*-OE | 0.176 | 89 | 0.200* | 0.965 | 89 | 0.096 |
|  | WT | 0.099 | 83 | 0.149 | 0.955 | 83 | 0.120 |
|  | *pcap2* | 0.247 | 84 | 0.096 | 0.914 | 84 | 0.086 |
|  | *PCaP2*-RNAi | 0.286 | 79 | 0.200* | 0.987 | 79 | 0.243 |
| Drought | *PCaP2*-OE | 0.257 | 78 | 0.200* | 0.886 | 78 | 0.366 |
|  | WT | 0.192 | 84 | 0.200* | 0.995 | 84 | 0.094 |
|  | *pcap2* | 0.281 | 87 | 0.116 | 0.868 | 87 | 0.288 |
|  | *PCaP2*-RNAi | 0.196 | 86 | 0.189 | 0.992 | 86 | 0.392 |
|  | | Leaf area | | | | | |
| CK | *PCaP2*-OE | 0.097 | 223 | 0.200* | 0.967 | 223 | 0.076 |
|  | WT | 0.149 | 217 | 0.200* | 0.927 | 217 | 0.543 |
|  | *pcap2* | 0.233 | 254 | 0.200* | 0.874 | 254 | 0.322 |
|  | *PCaP2*-RNAi | 0.165 | 218 | 0.168 | 0.921 | 218 | 0.127 |
| Drought | *PCaP2*-OE | 0.192 | 215 | 0.14 | 0.886 | 215 | 0.059 |
|  | WT | 0.172 | 216 | 0.200* | 0.974 | 216 | 0.138 |
|  | *pcap2* | 0.152 | 246 | 0.200* | 0.976 | 246 | 0.942 |
|  | *PCaP2*-RNAi | 0.187 | 225 | 0.200* | 0.902 | 225 | 0.121 |
|  | | Root hair length | | | | | |
| CK | WT | 0.134 | 251 | 0.124 | 0.949 | 251 | 0.088 |
|  | *pcap2* | 0.095 | 255 | 0.200* | 0.948 | 255 | 0.058 |
|  | *PCaP2*-RNAi | 0.086 | 253 | 0.200* | 0.968 | 253 | 0.169 |
| Drought | WT | 0.136 | 250 | 0.121 | 0.950 | 250 | 0.134 |
|  | *pcap2* | 0.098 | 232 | 0.200* | 0.962 | 232 | 0.303 |
|  | *PCaP2*-RNAi | 0.238 | 214 | 0.200* | 0.872 | 214 | 0.145 |

1. Lilliefors significant level correction.

*. This is the true minimum level of significance.

Abbreviations: Df, Degree of freedom.

**Supplementary Table S3** Chemicals and kits used in this study

| Murashige & Skoog (Sigma, cat. no. M0404) |
| --- |
| Phytoagar (PlantMedia, cat. no. 40100072-4) |
| X-gluc (Solarbio, cat. no. X8060) |
| PEG6000 (Solarbio, cat. no. P8250) |
| Abscisic Acid (Solarbio, cat. no. A8060) |
| Salicylic Acid (Solarbio, cat. no. S7080) |
| RNeasy Plant Mini Kit (Qiagen, cat. no. 74904) |
| RNeasy-Free DNase Set (Qiagen, cat. no. 79254) |
| Omniscript RT Kit (Qiagen, cat. no. 205111) |
| SuperReal PreMix Plus (SYBR Green) (TIANGEN, FP205) |
